# Radial peripapillary capillary density as a predictive factor for glaucoma in eyes with ocular hypertension. An observational, comparative, single-centred study

**DOI:** 10.12688/f1000research.140453.1

**Published:** 2023-11-10

**Authors:** Elpida Kollia, Evita-Evangelia Christou, Eleni Patsea, Styliani Alexia Papadonta, Dimitris Papaconstantinou

**Affiliations:** 1 Division of Ophthalmology, Faculty of Medicine, National and Kapodistrian University of Athens, Athens, Attica, Greece; 2 Faculty of Medicine, University Ophthalmology Clinic, University of Ioannina, Ioannina, Greece; 3 Glaucoma Department, Ophthalmiatreio Athenon, Athens, Attica, Greece; 4 Ophthalmology Department, St. Johannes Hospital Dortmund, Dortmund, Germany

**Keywords:** peripapillary capillary plexus, intraocular pressure, optical coherence tomography angiography, optic disc, glaucoma

## Abstract

**Background:** Ocular hypertension (OH) is a condition characterized by elevated intraocular pressure (IOP) exceeding the normal range, without any evident damage to the optic nerve or visual field defects characteristic of glaucoma. It constitutes a significant precursor to the development of glaucoma, a leading cause of irreversible vision loss worldwide. Emerging evidence has shown that microcirculation alterations in eyes with OH could serve as predicting factors to identify eyes at high risk for progression to glaucoma. In view of the above, the purpose of our study is to investigate microcirculation alterations of the radial peripapillary capillary plexus using optical coherence tomography angiography (OCT-A) in patients with ocular hypertension (OH).

**Methods:** A total of 192 eyes were included in this observational, comparative, single-centre study and were divided in two groups: OH eyes and healthy controls. OCT-A was performed to analyze microcirculation characteristics at the peripapillary area. Radial peripapillary capillary density was measured at the total area of the optic disc and at each separate region (superior, inferior, inside). The parameters of age, medical treatment for ocular hypertension, sex and retinal fiber layer thickness were evaluated.

**Results:** Total radial peripapillary capillary density was significantly lower in patients with OH than in healthy controls Concerning the microcirculation characteristics at each separate region of the peripapillary area, the results were as follows: inferior radial peripapillary capillary density was significantly decreased in individuals with OH than in controls, while measurements in the superior peripapillary area and internal optic disc were similar in both groups.

**Conclusions:** Our study indicates decreased radial peripapillary capillary density in eyes with OH. Microcirculation alterations in the inferior peripapillary area could potentially comprise biomarkers for OH progression to glaucoma.

## Introduction

Glaucoma is one of the leading causes of progressive visual loss worldwide. Despite advances in our understanding of the pathophysiology of glaucoma, the only proven efficacious intervention for preventing further progression of the optic nerve impairment includes lowering of intraocular pressure. ^1^^–^^4^ Although numerous risk factors have been identified (family history, age, central corneal thickness), elevated intraocular pressure (IOP) remains the key element for the development of glaucoma and the sole modifiable parameter at present. Patients with elevated IOP (>21mmHg in one or both eyes) and no signs of detectable glaucomatous damage in the standard clinical tests are considered to have ocular hypertension (OH). The prevalence of OH is estimated to range from 3–10% in the population over the age of 40 years, while approximately 10% of them may eventually develop glaucoma. This high incidence of OH and the resultant potential visual deterioration raises critical issues about identifying novel predicting factors and diagnostic tests to determine the appropriate patients who are at risk of developing glaucoma. ^1^^–^^5^^,^^8^^–^^10^

Neuronal degeneration is a firmly established characteristic that plays a central role in the development of glaucoma. Recent evidence has documented vasculature changes in the optic nerve head and the peripapillary retina in the context of this disorder highlighting the role of microcirculation dysfunction as a contributing factor to both the onset and progression of the disease. The radial peripapillary capillary plexus is a distinct vascular layer located in the retinal nerve fiber layer and provides oxygen and nourishment in the retinal ganglion cells. Indeed, the integrity of the optic nerve blood supply and the resultant structural neuronal vulnerability may have an intimate association in the pathogenesis of glaucoma.

Since the radial peripapillary capillary network could be primarily affected in primary open angle glaucoma (POAG) even before structural alterations occur, the investigation of validated microcirculation biomarkers for early detection, profound understanding of the pathogenesis and prompt treatment of glaucoma is a promising aspect. ^6^^–^^7^^,^^11^ Optical coherence tomography angiography (OCT-A) is a novel, non-invasive imaging technique that generates depth-resolved angiograms and enables visualization and quantification of blood flow alterations in the retina and optic nerve. Radial peripapillary capillary plexus parameters demonstrate considerable changes prior to neuronal degeneration in glaucomatous eyes, thus providing new insights in our understanding of the pathobiology. These emerging findings have fuelled recent research providing evidence that microcirculation alterations in eyes with OH could serve as predicting factors to identify eyes at high risk for progression to glaucoma.

In the present study, we sought to characterize microvasculature alterations of the peripapillary area in patients with OH. The aim of the study was to identify potential objective biomarkers that designate these eyes and contribute to thorough clinical assessment that may provide evidence of further progression to glaucoma. ^1^^,^^12^^–^^22^

## Methods

In individuals of the OH group, the mean values of radial peripapillary capillary density in the inferior area were significantly lower than those in healthy controls (p=0.025). The study was conducted between November 2019 and March 2020 at the Glaucoma Clinic of Ophthalmiatreion Athenon in Athens, Greece after receiving approval from the Ethics Committee of the National and Kapodistrian University of Athens (ID: 1819009698) (Date:08/11/2018) and adhered to the tenets of the declaration of Helsinki. Written informed consent was obtained from each patient so that their data could be used for research purposes.

The dates of recruitment were identical to the period of the study and the participants were not required to attend any follow-up appointments.

This was an observational, retrospective, comparative, single-centre study. In order for the sample size to be arrived, we recruited all patients who had a clinical diagnosis of OH. To be included, eyes had IOP> 21 mmHg and the optic nerve had no structural glaucomatous damage on spectral domain - optical coherence tomography angiography (SD-OCT). Patients that were under treatment with more than two IOP-lowering medications and had an increased risk for progression to primary open angle glaucoma (POAG) and those who had undergone selective laser trabeculoplasty (SLT) were excluded. ^1^^,^^2^ Furthermore, exclusion criteria consisted of retinal pathology or any other ocular or systemic comorbidity that could affect retinal vascular circulation, including diabetic and hypertensive retinopathy, retinal vein occlusion, uveitis or eyes with concomitant vitreoretinal pathology. Finally, we excluded patients if the OCT-A images were of low quality due to media opacities or motion artifacts.

Eyes were divided into two groups: eyes with OH and healthy controls. The control group comprised of healthy individuals with no ocular or systemic comorbidities.

A comprehensive ophthalmological examination was performed. The examination included anterior segment and dilated fundus examination with slit lamp biomicroscopy (on Haag-Streit Slit Lamp Imaging Module 910), visual acuity (LogMAR) and IOP measurement with Goldman applanation tonometer.

OCT was performed for each patient to exclude any glaucomatous damage. OCT-A of the optic nerve was obtained for all patients.

### OCT-A imaging protocols

The scanning area was captured using the AngioVue HD software (OptoVue, Fremont, CA, USA) using the split spectrum amplitude decorrelation angiography (SSADA). Eye tracking was employed to minimize motion artifacts, and for each study participant, an optic nerve head scan was obtained. Subsequently, all OCT-A scans underwent a thorough manual examination and correction of segmentation errors by two separate investigators. Additionally, the images were assessed for quality, taking into account factors such as artifacts impacting microvasculature analysis, motion artifacts, suboptimal fixation, and signal strength index (adequate quality was defined as a strength index of ≥7 out of 10).OCT-A imaging of the optic nerve head was acquired using a 4.5x4.5mm scan pattern. Radial peripapillary capillary plexus (RPC) flow density was measured automatically by the device software (Beam Spot Size: 22μm, Depth: 3.0μm digital resolution, Transverse: 2mm to 12mm, Scan Beam Wavelength: =840±10nm, Exposure Power at pupil: 750μW maximum).

Patients were divided into two groups due to the absence of normative data regarding the RPC density values: a patient group with eyes consistent with ocular hypertension (66.3%), and a control group (33.7%).

### Statistical analyses

All analyses were performed using SPSS statistical software (version 22.0) (RRID:SCR_002865). We used independent sample Student's t-tests to compare mean values between the control and patient groups. Quantitative variables are expressed as mean (SD) values, while qualitative variables are expressed as absolute and relative frequencies. Chi-squared tests were used to compare proportions. Multiple linear regression analysis was used with dependence on the total peripapillary capillary density (PP) values. The regression equation included parameters such as sex, age, and medical treatment for OH. Adjusted regression coefficients (β) with standard errors were computed from the results of the linear regression analyses. All reported P-values are two-tailed. Statistical significance was set at a P-value <0.05.

## Results

After applying exclusion criteria, a total of 192 eyes with OH were included in the analysis. The OH group comprised of 127 eyes (66.3%) and the control group of 65 eyes (33.7%). ^24^ The mean age was significantly higher in patients with OH (63.5 years) than in healthy controls (53.6 years) (p<0.001). Our study included more female participants as compared to men, though without significant differences between the OH and control group. The patients in the control group were not under treatment for any ocular disorder.

Demographic characteristics are shown in Table 1.

**Table 1.** Sample characteristics of each group (absolute and relative frequencies for qualitative characteristics, mean [standard deviation] for quantitative characteristics).

	Group		P-value
	Controls N = 65; 100%	Patients with ocular hypertension N = 127; 66.3%	
	N (%)	N (%)	
Age, mean (SD)	53.6 (14.7)	63.5 (11.6)	<0.001 ^+^
Sex			
Female	34 (59.6)	66 (58.9)	0.928 ^++^
Male	23 (40.4)	46 (41.1)	
Under medical treatment	0 (0.0)	56 (50.0)	<0.001 ^++^

^+^ Student’s t-test.

^++^ Pearson’s chi-squared test.

In individuals of the OH group, the mean values of radial peripapillary capillary density in the total area were significantly lower than those in healthy controls (p=0.041). Concerning the subgroup analysis in the superior, inferior and internal disc area, the results were as follows. In individuals of the OH group, the mean values of radial peripapillary capillary density in both the superior and internal optic disc area were lower, though not statistically significant, than those in healthy controls, while the mean values of radial peripapillary capillary density in the inferior area were significantly lower in individuals with OH than in controls (p=0.025). OCT-A parameters in both groups are shown in Table 2.

**Table 2.** Peripapillary capillary density (PP) measurements of each study group (mean [standard deviation] and p-value for Student’s t-test).

	Group		P ^+^
	Controls N = 65; 100%	Patients with ocular hypertension N = 127; 66.3%	
	Mean (SD)	Mean (SD)	
Total PP	52.3 (3.2)	51.1 (3.8)	0.041
Superior	52.4 (4.0)	51.4 (4.4)	0.145
Inferior	52.1 (3.4)	50.5 (4.9)	0.025
Inside disc	51.0 (6.4)	50.6 (6.1)	0.703

^+^ Student’s t-test used to calculate p-value.

While accounting for age, sex and medical treatment we found similar PP measurements in both control and OH group ( Table 3). In contrast, a correlation was found between the decrease of PP and age increase. In all measurements, men presented significantly lower PP values than women, while no noticeable association was found between medical treatment and PP values.

**Table 3.** Multiple linear regression analysis results with peripapillary capillary density (PP) measurements as dependent variables and group, age, sex, and medical treatment as independent variables (regression coefficients [β] and standard errors (SE)).

	Total PP		Superior		Inferior		Inside disc	
	β (SE) ^+^	P	β (SE) ^+^	P	β (SE) ^+^	P	β (SE) ^+^	P
Group								
Controls (reference)								
Patients with ocular hypertension	−0.07 (0.67)	0.920	−0.04 (0.8)	0.960	−0.17 (0.85)	0.844	0.58 (1.21)	0.630
Age	−0.09 (0.02)	**<0.001**	−0.09 (0.02)	**<0.001**	−0.08 (0.03)	**0.004**	−0.07 (0.04)	0.078
Sex (reference)								
Female								
Male	−1.77 (0.53)	**0.001**	−2.14 (0.64)	**0.001**	−1.73 (0.67)	**0.011**	−2.6 (0.96)	**0.008**
Medical treatment								
No (reference)								
Yes	−0.56 (0.64)	0.382	−0.16 (0.77)	0.837	−1.42 (0.81)	0.081	−0.61 (1.16)	0.603

^+^ Regression coefficient (standard error).

## Discussion

The current gold standards for establishing a diagnosis for glaucoma include clinical features of glaucomatous damage to the optic nerve (deepening of excavation and bleeding of optic nerve), elevated IOP and morphological changes in structural OCT (defects of the retinal nerve fiber layer). OH consists of the sole modifiable parameter that delays progression of degeneration of the optic nerve. ^1^^,^^3^^–^^10^ Objective biomarkers for identifying individuals with OH that are at risk for developing glaucoma prior to apparent neurodegeneration occurs consist a valuable and unmet need. In this aspect, recent evidence has documented microvasculature alterations in the context of OH. Considering that impaired neuronal function is a widely recognized concern among individuals with glaucoma, it is valuable to assess the potential pathophysiological underpinnings of vascular decline as a predicting factor for patients with OH who are at risk for developing glaucoma. In view of the above, by implementing OCT-A, we sought to assess microvasculature alterations affecting the radial peripapillary capillary plexus in patients with OH in order to identify differences from healthy eyes. Our study focuses on topographical changes of microcirculation in the peripapillary area. Our results indicate that alterations of the peripapillary capillaries may comprise a potential biomarker of OH progression to glaucoma.

The Ocular Hypertension Treatment Study (OHTS) was a longitudinal trial. Besides assessing the impact of treatment on individuals with ocular hypertension (IOP ranging from 24 mmHg to 32 mmHg), the study provided valuable insights into various potential factors contributing to the transition from OH to glaucoma.

For ocular hypertension, validated predictive models utilize an individual's ocular parameters to ascertain the likelihood of developing glaucoma. ^1^^,^^3^^–^^5^^,^^8^^–^^11^

The risk factors and conditions pertaining to OH have been thoroughly analyzed in various studies. ^1^^–^^9^ Critical features predictive of POAG include older age, race (African American), sex (male), larger vertical cup-disc ratio, larger horizontal cup-disk ratio, increased IOP, higher Humphrey visual field pattern standard deviation (SD), decreased retinal nerve fibre layer (RNFL) values, heart disease, and thinner central cornea. ^1^^,^^3^^,^^4^^–^^10^

In this study, we assessed microvasculature alterations in the peripapillary area, especially in total and in each separate region of the optic nerve; superior, inferior and internal area. Our results indicate that the capillary plexus density was decreased in the total peripapillary area in OH eyes as compared to healthy individuals. Interestingly, evaluation of retinal blood flow in patients with OH has indicated that the typically robust peripapillary microvascular network shows a reduction in both the superficial disc vasculature and the deep lamina cribrosa. Furthermore, lower PP values may be associated with the decreased RNFL measurements that are usually found in OH. Therefore, RNFL thickness and RPC density may be related to subsequent glaucomatous defects in the optic nerve head. ^1^^,^^16^^–^^22^ Concerning the analysis of each separate region of the optic nerve, our results indicate variability. In particular, capillary plexus density in the inferior peripapillary area was decreased in OH eyes as compared to healthy controls, while the superior and inside the optic disc microcirculation was similar between the two groups. These changes may be associated with a predisposition to future structural changes in optic nerve head. The aforementioned hypothesis is in line with the “ISNT” rule that has been widely used in clinical practice as an early screening tool in terms of assessing the optic nerve head appearance. ^1^^,^^3^^,^^4^^,^^23^ Indeed, a potential correlation between RPC changes in the inferior part of the disc and the widely used “ISNT” pattern (inferior, superior, nasal, temporal), referring to the normal optic disc rim width (inferior ≥ superior ≥ nasal ≥ temporal) may support evidence indicating possible OH conversion to POAG in these eyes. Overall, our results suggest that the inferior radial peripapillary capillary plexus alterations could be a biomarker for predisposition to structural changes of the optic disc, which in turn might be an indicator of ocular hypertension progression to glaucoma.

According to the OHTS men are more likely to progress to primary open-angle glaucoma (POAG). In our study we found that men had consistently reduced PP measurements compared to women. Thus, this could also be considered as a factor predictive of POAG. ^1^^,^^3^^–^^5^

To date, a number of studies have shown evidence for microvasculature alterations in the peripapillary area in eyes with OH. ^6^^–^^7^ We consider this information highly relevant for clinical practise as blood changes could serve as a predicting factor for the progression of OH to glaucoma, even before apparent structural neuronal changes. Identifying eyes who are at risk for disease progression would optimise the outcomes for this subgroup of patients as well as provide evidence for treatment options and prognostication. The aforementioned parameter along with the large cohort of patients that were recruited may add strength to our study. In any case, our study contributes to a topic which warrants further investigation in clinical practice. Inevitably, certain limitations should be considered. Firstly, in addition to peripapillary capillary plexus vessel density, further vascular parameters should be analyzed in order to have a more detailed and comprehensive investigation. Notwithstanding the fact that the total peripapillary area seems to be mainly affected, with the inferior part of the optic nerve to indicate changes primarily, a proper correlation with the OCT structural changes would add value to similar studies. Moreover, our control group was not age-matched and one could support that physiologic differences among age groups could have contributed to the difference in vascular parameters, however this has not been proven in clinical practice. Lastly, we should consider the intrinsic limitations of the OCT-A technology and the imaging artifacts that could interfere with precise examination of the angiograms.

In conclusion, this study points out that peripapillary capillary plexus alterations in eyes with OH indicate a close association with the known risk factors for OH progression to glaucoma. Thus, on this basis we could assume that microcirculation of the optic nerve may provide insight into further morphological changes and that could differentiate healthy eyes from those with OH comprising potential predicting factor for the prompt diagnosis of glaucoma. The latter comprises a pivotal finding which could be used as a unique screening tool for glaucoma specialists. Additional prospective longitudinal studies are needed to validate our findings and determine the accuracy of OCT-A parameters as predicting factors in clinical practice.

## Data Availability

Mendeley Data: Radial peripapillary capillary density as a predictive factor for glaucoma in eyes with ocular hypertension. https://doi.org/10.17632/h9tntd4gkf.1. ^24^ This project contains the following underlying data:
• DATA.xlsx Data are available under the terms of the Creative Commons Attribution 4.0 International license (CC-BY 4.0).

## References

[1] Kollia E : Correlation Between Central Corneal Thickness and Radial Peripapillary Capillary Density, in Patients with Ocular Hypertension. *Cureus.* 2021 Aug 12;13(8):e17138. eCollection 2021 Aug. 10.7759/cureus.17138 34408962 PMC8362868

[2] Inner retinal layers' alterations of the microvasculature in early stages of Parkinson's disease: a cross sectional study. Evita Evangelia Christou et al. *Int Ophthalmol.* 2023 Jul;43(7):2533–2543. Epub 2023 Mar 4. 10.1007/s10792-023-02653-x 36869977

[3] Salmon JF : *Glaucoma. Kanski’s Clinical Ophthalmology: A Systematic Approach.* 9th ed. Edinburgh, Scotland: Elsevier;2020. 978-0-7020-7713-5. OCLC 1131846767.

[4] European Glaucoma Society: EGS Guidelines, 5th edition.

[5] Bhatti MT : *American Academy of Ophthalmology: 2019-2020 BCSC: Basic and Clinical Science Course.* San Francisco: American Academy of Ophthalmology;2019.

[6] Radial Peripapillary Capillary Network Visualized Using Wide-Field Montage Optical Coherence Tomography Angiography. 10.1167/iovs.15-18877 27454659

[7] Al-Nashar HY El-Haig WM Al-Naimy MA : Assessment of radial peripapillary capillary and macular vascular density in primary open angle glaucoma. *J. Egypt. Ophthalmol. Soc.* 113:46. 10.4103/ejos.ejos_11_20

[8] Gordon MO Beiser JA Brandt JD : The Ocular Hypertension Treatment Study: baseline factors that predict the onset of primary open-angle glaucoma. *Arch. Ophthalmol.* 2002;120:714–720. 10.1001/archopht.120.6.714 12049575

[9] Lee BL Wilson MR : Ocular Hypertension Treatment Study (OHTS) commentary. *Curr. Opin. Ophthalmol.* 2003;14:74–77. 10.1097/00055735-200304000-00003 12698045

[10] Gordon MO Kass MA : The Ocular Hypertension Treatment Study: design and baseline description of the participants. *Arch. Ophthalmol.* 1999;117:573–583. 10.1001/archopht.117.5.573 10326953

[11] Kass MA : The Ocular Hypertension Treatment Study. A randomized trial determines that topical ocular hypotensive medication delays or prevents the onset of primary open-angle glaucoma. *Arch. Ophthalmol.* 2002;120:701–713. 10.1001/archopht.120.6.701 12049574

[12] Kashani AH Chen CL Gahm JK : Optical coherence tomography angiography: A comprehensive review of current methods and clinical applications. *Prog. Retin. Eye Res.* 2017;60:66–100. 10.1016/j.preteyeres.2017.07.002 28760677 PMC5600872

[13] Alnawaiseh M Lahme L Eter N : Optische Kohärenztomographie-Angiographie: Stellenwert in der Glaukomdiagnostik [Optical coherence tomography angiography: Value for glaucoma diagnostics]. *Der. Ophthalmologe.* 2019;116:602–609. 10.1007/s00347-018-0815-9 30413870

[14] Akil H Falavarjani KG Sadda SR : Optical coherence tomography angiography of the optic disc; an overview. *J. Ophthalmic. Vis. Res.* 2017;12:98–105. 10.4103/2008-322X.200162 28299012 PMC5340069

[15] Yip VCH Wong HT Yong VKY : Optical coherence tomography angiography of optic disc and macula vessel density in glaucoma and healthy eyes. *J. Glaucoma.* 2019;28:80–87. 10.1097/IJG.0000000000001125 30461553

[16] Jia Y Wei E Wang X : Optical coherence tomography angiography of optic disc perfusion in glaucoma. *Ophthalmology.* 2014;121:1322–1332. 10.1016/j.ophtha.2014.01.021 24629312 PMC4082728

[17] Chihara E Dimitrova G Amano H : Discriminatory power of superficial vessel density and prelaminar vascular flow index in eyes with glaucoma and ocular hypertension and normal eyes. *Investig. Ophthalmol. Vis. Sci.* 2017;58:690–697. 10.1167/iovs.16-20709 28134965

[18] Lauermann JL Eter N Alten F : Optical coherence tomography angiography offers new insights into choriocapillaris perfusion. *Ophthalmologica.* 2018;239:74–84. 10.1159/000485261 29353272

[19] Van Melkebeke L Barbosa-Breda J Huygens M : Optical coherence tomography angiography in glaucoma: a review. *Ophthalmic. Res.* 2018;60:139–151. 10.1159/000488495 29794471

[20] Ozcan Y Ozcaliskan S Balci S : The correlation of radial peripapillary capillary density measurements with optic nerve head morphology and retinal nerve fiber layer thickness in healthy eyes. *Photodiagnosis Photodyn Ther.* 2020;32:102008. 10.1016/j.pdpdt.2020.102008 32942046

[21] Miguel A : OCT-angiography detects longitudinal microvascular changes in glaucoma: a systematic review. 106:667–675. 10.1136/bjophthalmol-2020-318166 33452184

[22] Monteiro-Henriques I Rocha-Sousa A Barbosa-Breda J : Optical coherence tomography angiography changes in cardiovascular systemic diseases and risk factors: A Review. 100. 10.1111/aos.14851 33783129

[23] Moon J Park KH Kim DM : Factors affecting ISNT rule satisfaction in normal and glaucomatous eyes. *Korean J. Ophthalmol.* 2018;32:38–44. 10.3341/kjo.2017.0031 29376225 PMC5801088

[24] Kollia E : Radial peripapillary capillary density as a predictive factor for glaucoma in eyes with ocular hypertension.[Dataset]. *Mendeley Data.* 2023;V1. 10.17632/h9tntd4gkf.1 PMC10755262 38161427

