# Supplementary material for: Radial peripapillary capillary density as a predictive factor for glaucoma in eyes with ocular hypertension. An observational, comparative, single-centred study
Source: F1000Res. 2023 Nov 10;12:1456. [Version 1] doi: 10.12688/f1000research.140453.1 (PMC10755262; doi:10.12688/f1000research.140453.1)
Supplement: Peer review history

# Reviewer report: Reviewer response for version 1

**DOI:** 10.5256/f1000research.153805.r229783

**Published:** 2024-01-25

**Authors:** Anastasios Sepetis

**Affiliations:** 1 University Hospital Southampton NHS Foundation Trust, Southampton SO, UK

The study provides valuable insights into the potential role of radial peripapillary capillary density as a biomarker of ocular hypertension and glaucoma.

 The control group is not age-matched with the OHT group, which could introduce bias as there is inverse correlation of RPC density with age. 

 Please rephrase verb arrived from the sentence: "In order for the sample size to be arrived, we recruited all patients who had a clinical diagnosis of OH."

Is the work clearly and accurately presented and does it cite the current literature?

Yes

If applicable, is the statistical analysis and its interpretation appropriate?

I cannot comment. A qualified statistician is required.

Are all the source data underlying the results available to ensure full reproducibility?

Yes

Is the study design appropriate and is the work technically sound?

Yes

Are the conclusions drawn adequately supported by the results?

Partly

Are sufficient details of methods and analysis provided to allow replication by others?

Yes

Reviewer Expertise:

Glaucoma

I confirm that I have read this submission and believe that I have an appropriate level of expertise to confirm that it is of an acceptable scientific standard.

# Reviewer report: Reviewer response for version 1

**DOI:** 10.5256/f1000research.153805.r229781

**Published:** 2024-01-02

**Authors:** Kirsten Julia Habbe

**Affiliations:** 1 St. Johannes Hospital, Dortmund, DEU, Germany

Peer Review Report

 Title: "Radial Peripapillary Capillary Density as a Predictive Factor for Glaucoma in Eyes with Ocular Hypertension: An Observational, Comparative, Single-Centered Study"

 The article investigates a crucial aspect of glaucoma prediction in eyes with ocular hypertension, and it presents a well-structured observational study. The findings have the potential to contribute significantly to our understanding of early glaucoma indicator.

 I have no further queries concerning this article.

Is the work clearly and accurately presented and does it cite the current literature?

Yes

If applicable, is the statistical analysis and its interpretation appropriate?

Yes

Are all the source data underlying the results available to ensure full reproducibility?

Yes

Is the study design appropriate and is the work technically sound?

Yes

Are the conclusions drawn adequately supported by the results?

Yes

Are sufficient details of methods and analysis provided to allow replication by others?

Yes

Reviewer Expertise:

glaucoma

I confirm that I have read this submission and believe that I have an appropriate level of expertise to confirm that it is of an acceptable scientific standard.

# Reviewer report: Reviewer response for version 1

**DOI:** 10.5256/f1000research.153805.r229782

**Published:** 2023-12-28

**Authors:** Anastasios Lavaris

**Affiliations:** 1 Moorfields Eye Charity, London, England, UK; 2 National and Kapodistrian University of Athens, Athens, Greece

I had the opportunity to review this manuscript and I am pleased to provide a positive assessment of this work.

 It is a well designed and presented study on the role of peripapillary capillary density as a predictive factor for glaucoma in eyes with ocular hypertension.

 There is logical flow of information, from introduction to methodology and results and that contributes to the overall readability.

 The inclusion of up-to-date references further enhances the credibility and relevance of the content.

 My only criticism is the fact that the first sentence of 'Methods' section is not relevant and should be moved in the 'Results' section. That should be amended by the authors prior to acceptance.

 In conclusion, only this minor flaw has to be corrected.

Is the work clearly and accurately presented and does it cite the current literature?

Yes

If applicable, is the statistical analysis and its interpretation appropriate?

Yes

Are all the source data underlying the results available to ensure full reproducibility?

Yes

Is the study design appropriate and is the work technically sound?

Yes

Are the conclusions drawn adequately supported by the results?

Yes

Are sufficient details of methods and analysis provided to allow replication by others?

Yes

Reviewer Expertise:

Ophthalmology with a special interest in glaucoma

I confirm that I have read this submission and believe that I have an appropriate level of expertise to confirm that it is of an acceptable scientific standard.

# Reviewer report: Reviewer response for version 1

**DOI:** 10.5256/f1000research.153805.r224087

**Published:** 2023-12-22

**Authors:** Panagiotis Sergouniotis

**Affiliations:** 1 University of Manchester, Manchester, UK

This interesting study assessed if individuals with ocular hypertension have differences in the microcirculation of their radial peripapillary capillary plexus compared to unaffected individuals. A reasonably large sample size was available and a helpful file with the underlying data is included.

 I have two main points and a few minor suggestions

 MAIN POINTS:

 ** Referencing and discussion of previous literature can be improved. I have highlighted below a few relevant sections. The main recommendation though is to include a few more sentences in the Discussion after the statement that "To date, a number of studies have shown evidence for microvasculature alterations in the peripapillary area in eyes with OH. ^6^ ^– 7^." For example, additional information on what these studies have shown would be of interest.

 ** It would be helpful to elaborate a bit on the interpretation of the results of the linear regression analysis. Would these suggest that the study findings should be interpreted more cautiously?

 MINOR SUGGESTIONS

 Introduction

 ** Please include references supporting the statements in the second paragraph

 Methods

 ** The first sentence ("In individuals ... controls (p=0.025)") would be more appropriate for the Results section. Also, in this phrase and in other sentences in the Abstract and in the Results, the findings are described as significant. It would be less subjective and more precise to describe them as statistically significant (especially given that the differences between groups are small which makes it challenging to conclude about clinical significance)

 ** Did the analysis focus on the left, the right, or the mean between eyes?

 ** It is mentioned that "Radial peripapillary capillary plexus (RPC) flow density was measured " but in the next subsection, it is stated that "Multiple linear regression analysis was used with dependence on the total peripapillary capillary density (PP) values." It would be helpful to clarify (for the non expert) what was the parameter/metric that was evaluated (is RPC flow density the same as PP?). It would also be worth considering minimising abbreviations and, potentially, including an illustrative image.

 Results

 ** Not necessary but did the research team collect data on the refractive error (or axial length) of study participants?

 **  I assume that the primary analysis focused on total peripapillary capillary density and that the subgroup analyses in the superior, inferior and internal disc areas were secondary studies. could it be appropriate to apply multiple comparison correction (FDR or Bonferroni) for the 3 secondary analyses?

 ** In Table 2, one of the headers mentions "Controls N = 65; 100%" while the other mentions "Patients with ocular hypertension N = 127; 66.3%". Should the former have 33.7% instead of 100%?

 ** Would it be worth considering including a plot with the linear regression findings?

 Discussion

 ** Please consider amending the following slightly suboptimal sentence "Objective biomarkers for identifying individuals with OH that are at risk for developing glaucoma prior to apparent neurodegeneration occurs consist a valuable and unmet need."

 ** Please add references to substantiate the statement that "In this aspect, recent evidence has documented microvasculature alterations in the context of OH."

 ** Please include OHTS reference.

 ** Paragraphs 2, 3 and 4 (from "The Ocular ..." to "central cornea") could be merged and rephrased to enhance the clarity and coherence of the text.

 References

 ** Referencing is not standardised and key information (e.g. year of publication) is missing from a number of references (e.g. 5 and 6).

Is the work clearly and accurately presented and does it cite the current literature?

Yes

If applicable, is the statistical analysis and its interpretation appropriate?

Partly

Are all the source data underlying the results available to ensure full reproducibility?

Yes

Is the study design appropriate and is the work technically sound?

Yes

Are the conclusions drawn adequately supported by the results?

Yes

Are sufficient details of methods and analysis provided to allow replication by others?

Yes

Reviewer Expertise:

Vision research, retinal imaging, bioinformatics

I confirm that I have read this submission and believe that I have an appropriate level of expertise to confirm that it is of an acceptable scientific standard, however I have significant reservations, as outlined above.

# Reviewer report: Reviewer response for version 1

**DOI:** 10.5256/f1000research.153805.r224092

**Published:** 2023-12-07

**Authors:** Susmito Biswas

**Affiliations:** 1 Manchester Royal Eye Hospital, Manchester, UK

This is a study comparing the retinal peripapillary capillary (RPC) plexus vessel density and intra-disc vessels density in subjects with ocular hypertension (OHT) versus controls (no history of OHT) using optical coherence tomography angiography (OCTA). The study's main findings are:

1. The overall RPC density in OHT subjects were lower than controls.
2. The inferior quadrant RPC density was significantly lower than controls but other quadrants were not significantly different and the intra-disc density was not significantly different.
3. There was a significant inverse correlation of RPC density with age, and correlation of reduced RPC density with male gender versus female gender.

The authors consider that reduction in RPC may be a biomarker of risk of progression of OHT to glaucoma.

 The main concerns with the paper at present are the following issues:

1. There is an inverse correlation of RPC density with age and there is a difference in age between OHT patients (older) and control patients (younger). Although this is mentioned in the discussion, the statement in the last paragraph of page 6 of the discussion where age is discussed, where the authors state "however this has not been proven in clinical practice" does need some reference to clarify this point.
2. The Retinal nerve fibre layer thickness is measured in OHT subjects but the paper does not comment in correlation of RPC with this nor does it measure RNFL in control subjects. RNFL thickness may reduce in glaucoma subjects and in OHT subjects that are converting to glaucoma and it is not clear if RPC density follows RNFL thinning or precedes RNFL thinning.
3. Other exclusion factors should be considered including abnormal axial length or high refractive errors, presence of narrow or occludable angles.
4. There may be variation in test and retest and diurnal variation of blood flow, so measurements taken at different times could alter RPC density. Did the authors capture one set of images per patient or more than one to give an average value from at least 2 sets of images per patient?
5. The manual segmentation of the images were done by investigators - please state whether or not they were masked to the group the patient belonged to. 

Is the work clearly and accurately presented and does it cite the current literature?

Yes

If applicable, is the statistical analysis and its interpretation appropriate?

I cannot comment. A qualified statistician is required.

Are all the source data underlying the results available to ensure full reproducibility?

Yes

Is the study design appropriate and is the work technically sound?

Yes

Are the conclusions drawn adequately supported by the results?

Partly

Are sufficient details of methods and analysis provided to allow replication by others?

Yes

Reviewer Expertise:

Paediatric ophthalmology

I confirm that I have read this submission and believe that I have an appropriate level of expertise to confirm that it is of an acceptable scientific standard, however I have significant reservations, as outlined above.
